# Temperance in the Army

**Published:** 1899-06-10

**Authors:** 


### Temperance in the Army.

The very interesting and important meetings held recently at the Royal United Service Institution and at the Westminster Town Hall, under the auspices of the Army Temperance Association, afforded opportunities for making known a very remarkable record of the good work which this Association has itself accomplished, or, in common with other conditions tending in the same direction, has at least very powerfully promoted. At the afternoon meeting Major-General Trotter, commanding the Home District, was able to say what universal experience will, we think, fully confirm, that it is now very rare to see in the streets of London a soldier the worse for drink; and Sir George White, who, in the absence of the Commander-in-Chief, took the chair at both meetings, was able to adduce some remarkable statistical evidence showing that the practice of temperance in India has been closely connected with a great diminution of military offences. The Marquis of Lansdowne, who spoke at the evening meeting, dwelt forcibly upon the incalculable importance of health to the soldier, as the first element of fitness for the discharge of his duty; and pointed out the greater power of the temperate to resist the various causes of disease to which soldiers on a campaign are exposed, as well as the diminished liability to the diseases of barrack life which is incidental to the exercise of self-control, whether in drink or in other forms of indulgence. He expressed his hope that the Army might not only be recruited for the future from the very best of the young lads of the country, but also that it should give them back to civil life all the better for the years which they had spent in the service. Nothing is more likely to conduce to the fulfilment of this hope than an increase of temperance in the ranks. We are not total abstainers, and cannot yield assent to the doctrines of those who would prohibit the reasonable and moderate use of alcohol. But we are convinced that strong and healthy young men, obtaining an abundance of nourishing food, do not require alcohol in any form, and that, in most circumstances, they are better without it; while in the classes from which the rank and file of the Army are mainly drawn it is probably far more easy to practise abstinence than temperance.

What may be called military literature, even the recent writings of so capable a person as Mr. Rudyard Kipling, are greatly too full of the convivial or the humorous, as opposed to the degrading, side of alcoholic excess; and abstinence is probably a better protection than mere temperance against the subtle influence of such ballads as "The Jacket" or "The Shut-Eye Sentry," with the "drippin' tight" of the former, and the captain with his corkscrew of the latter. We gather from the reports of the meetings that a serious difficulty is sometimes put in the way of military abstainers by the want of separate barrack-rooms for their use; and the Marquis of Lansdowne explained that, on this question, there was not the same unanimity everywhere as in the room in which he spoke. But, on the part of the War Office, he promised that in every new barrack there should be recreation-rooms from which intoxicating liquor was excluded; and it is not too much to expect that such a provision, by smoothing the road of the abstainer, and by removing some of the difficulties which now beset his path, will before long be justified by a better average of conduct and efficiency in new barracks than in old ones. If this expectation were realised, it would immensely strengthen the hands of the Temperance Association in asking for similar provision in the latter as well; and the cost of such provision, even if it were considerable, would not be grudged by a nation which is learning to be justifiably proud of its Army, and which is desirous that every reasonable advantage should be afforded to the men upon whom, after all, the national power and prosperity must in the last resource be dependent.

